# Evaluation of Lipidomics Profile of Quinoa Flour and Changes during Storage Based on Ultra Performance Liquid Chromatography Coupled with Quadrupole Exactive Orbitrap Mass Spectrometry

**DOI:** 10.3390/foods12244434

**Published:** 2023-12-11

**Authors:** Ya-bo Ba, Rui Li, Jia-yi Zhang, Liang Zou, Ding-Tao Wu, Yi-chen Hu

**Affiliations:** 1 Key Laboratory of Coarse Cereal Processing, Ministry of Agriculture and Rural Affairs, School of Food and Biological Engineering, Sichuan Engineering & Technology Research Center of Coarse Cereal Industrialization, Chengdu University, Chengdu 610106, China; 2 Institute for Advanced Study, Chengdu University, Chengdu 610106, China

**Keywords:** quinoa flour, lipase, storage oxidation, lipidomics, UPLC-Q-Exactive Orbitrap Mass spectrometry

## Abstract

Although quinoa is nutritious, its high fat content and lipase activity make it easily oxidized during storage. Meanwhile, quinoa’s lipid composition and changes during storage are still unknown. Therefore, we stored fresh quinoa flour at low temperature and low humidity (LL), normal temperature and normal humidity (NN), and high temperature and high humidity (HH) conditions for 120 days to assess its oxidative stability and to monitor the changes in lipid composition. Herein, the contents of fatty acids, the peroxide values, the malondialdehyde values, and the lipase activity in quinoa flour during storage are determined to evaluate its oxidation stability. At LL and NN conditions, the contents of fatty acids, the peroxide values, the malondialdehyde values, and the lipase activity changed slowly. They were 3 (LL) and 5 times (NN), 2.7 (LL) and 4.7 times (NN), 1.4 (LL) and 2.3 times (NN), and 1.5 (LL) and 1.6 times (NN) the initial content at storage up to 120 d. However, with the prolongation of storage time under HH conditions, they all increased significantly to 8, 6.6, 3, and 2 times the original content. Moreover, during the storage of quinoa under LL, NN, and HH conditions for 120 days, we continuously monitored the lipid composition of quinoa grains with UPLC-Q-Exactive Orbitrap MS/MS. We identified a total of 14 subclasses of 229 lipids, including 90 significantly different lipid species. PCA and PLS-DA showed that quinoa lipids in HH conditions changed significantly with prolonged storage; among these, the TG and DG classes were the most susceptible to oxidation, which could distinguish fresh quinoa from oxidized quinoa. Simultaneously, we also found that lipase activity has a significant impact on lipid metabolism through correlation analysis, which also indicates that enzyme inactivation treatment can slow down lipid hydrolysis and oxidation during storage. To explore the mechanism of these changes, we also identified twelve important lipid metabolism pathways during quinoa storage. In conclusion, our study advances knowledge of the storage stability and lipid oxidation mechanisms of quinoa and provides a theoretical basis for setting the shelf life of quinoa.

## 1. Introduction

Quinoa (*Chenopodium quinoa* Willd.) is a pseudocereal native to the Andean regions of South America, covering territories in Peru, Bolivia, Ecuador, Chile, and Argentina [1]. Quinoa has been recognized as a complete nutritional food. It is rich in protein (12–23%), dietary fibers (7–12%), and minerals (2.6 ± 0.2 g/100 g), and it contains a wide range of vitamins and a balanced range of essential amino acids [1]. In addition, quinoa flour has good gelation, water absorption, and emulsification properties, which are suitable for partially or completely replacing wheat flour in gluten-free products [2]. In our previous research, we found that the fat content in quinoa is relatively higher than that in other grains. The fat rate of quinoa is 6–7 g/100 g [3], with a high percentage of unsaturated fats. The composition of fatty acids (FAs) is similar to that of soy and corn grain. It is mainly composed of linoleic acid (49.0–56.4%), followed by oleic acid (19.7–29.5%) and linolenic acid (8.7–11.7%) [4]. Quinoa also has similar amounts of linolenic acid to soybean oil [5]. According to the latest statistics from the Food and Agriculture Organization of the United Nations, the yield of quinoa has increased significantly in recent years. In 2021, quinoa production in major producing areas, such as Peru, Ecuador, and Bolivia, increased to 147,037 tons. At the same time, much attention has also been paid to the addition of a certain amount of quinoa flour to produce gluten-free foods, such as cookies, bread, noodles, and others [2].

However, grinding quinoa during processing and storage increases its surface area and activates lipases, which accelerate the oxidative deterioration of quinoa. Wang et al. suggested that the lipase activity of buckwheat flour is associated with lipid degradation [6]. Lipid oxidation rancidity would produce a detrimental influence on the sensory quality, safety concerns, and nutritional and economic worth of foods. In the process of lipid oxidation, hydroxides are produced by enzymatic hydrolysis, enzymatic oxidation, and non-enzymatic oxidation. And then it emits a peculiarly pungent flavor and reduces sensory acceptance when the hydroxides decompose into aldehydes, ketones, alcohols, acids, and other low-molecular-weight carbonyl compounds, which decrease its nutritional quality and economic value [7]. In addition to causing bad flavors and color changes, lipid oxidation also destroys lipophilic bioactive compounds including carotenoids, phytosterols, and polyunsaturated fatty acids (PUFAs) [8]. At the same time, the process of lipid oxidation generates a large number of free radicals, and “Free radicals” have a greater effect on the body than nutrient loss and small volatile molecules because they can accelerate aging [9]. In addition, the long-term consumption of oxidized oils can lead to an unhealthy plasma lipid profile and cell failure, which further contributes to a wide range of diseases (cardiovascular diseases such as vascular inflammation and atherosclerosis) [10]. Therefore, determining lipid changes during storage and their relationship to lipase activity is very important for the application of quinoa flour in the food industry.

In recent years, with the constant development and application of histological technologies, high-throughput lipidomics as a new technology has also been widely used in various studies. Lipid molecules in specific tissues or organisms can usually be comprehensively analyzed and identified on a large scale to reveal their nutritional properties, physiological roles, and pathological mechanisms [11]. Lipidomics has also been widely employed in various studies on food. Based on their chemical functions and biochemical properties, lipids can be generally classified into eight classes, including fatty acids (FAs), glycerolipids (GLs), glycerophospholipids (GPs), sterol lipids (STs), sphingolipids (SPs), saccharolipids (SLs), prenol lipids (PRs), and polyketides (PKs), which encompass a total of 43,413 lipid species [12]. However, there have been very few studies to assess the changes in lipid composition and associated metabolic pathways during quinoa flour oxidation by lipidomics.

Therefore, the aim of this study was to evaluate the storage stability of quinoa flour and completely comprehend its lipid composition and the changes in lipid groups during storage. We prepared three varieties of quinoa flour with different storage conditions, including LL (low temperature and low humidity condition), NN (normal temperature and normal humidity condition), and HH (high temperature and high humidity condition). Firstly, we assessed oxidative stability using the content of fatty acids, the peroxide value, malondialdehyde value, and lipase activity. Secondly, the lipid composition of quinoa flour and fluctuations during storage were comprehensively characterized by UPLC-Q-Exactive Orbitrap MS/MS. At the same time, multivariate statistical analysis was performed to analyze the relationship between lipid changes and the lipase activity of quinoa flour during storage. These findings give us a better understanding of quinoa’s storage stability and provide a theoretical basis for setting a shelf life.

## 2. Materials and Methods

### 2.1. Materials

Fresh quinoa seeds (ZL-7) used in this study were collected from Sichuan province of China in harvest season. Following the cleaning and low-temperature air drying at 40 °C steps, the collected samples were dehulled, grinded into powder, and passed through an 80-mesh sieve. Then, the quinoa flour was placed in three colorless transparent polythene bags, which were oxidized for 120 d in three incubators. The storage was performed under low temperature and low humidity conditions (LL, 5 ± 2 °C, relative humidity 50 ± 2%), normal temperature and normal humidity conditions (NN, 25 ± 2 °C, relative humidity 65 ± 2%), and high temperature and high humidity conditions (HH, 40 ± 2 °C, relative humidity 80 ± 2%), respectively. It was possible to move their position in the incubator, as well as shake them once every 24 h. Then, oxidized quinoa flour samples were collected every 30 days and kept in a colorless transparent polythene bag at −80 °C until further analysis. (This was only used to save samples in different periods for subsequent unified analysis). Finally, lipid information was collected from all samples (0, LL30, LL60, LL90, LL120, NN30, NN60, NN90, NN120, HH30, HH60, HH90, HH120).

### 2.2. Chemicals and Reagents

High-performance liquid chromatography (HPLC)-grade acetonitrile (ACN) and methanol (MeOH) were supplied by Chengdu Kelong Chemical Reagents Company (Chengdu, China). HPLC-grade isopropanol (IPA), methyl tert-butyl ether (MTBE), and Ammonium acetate (>99.0%) were purchased from Shanghai Aladdin Biochemical Technology Company (Shanghai, China).

All internal standards were purchased from Avanti Polar Lipids (Alabaster, AL, USA) and prepared as mixed solution in MeOH, including triacylglycerol (d_7_-TG-15:0/18:1, 55 μg·mL^−1^), diacylglycerol (d_7_-DG-15:0/18:1, 12 μg·mL^−1^), phosphatidylcholine (d_7_-PC-15:0/18:1, 160 μg·mL^−1^), phosphatidylethanolamine (d_7_-PE-15:0/18:1, 5 μg·mL^−1^), phosphatidylserine (d_7_-PS-15:0/18:1, 8 μg·mL^−1^), phosphatidylinositol (d_7_-PI-15:0/18:1, 8 μg·mL^−1^), cholesterol ester (CE-18:1, 350 μg·mL^−1^), sphingomyelin (d_9_-SM-d18:1/18:0, 30 μg·mL^−1^), lysophosphatidylethanolamine (d_7_-Lyso PE-18:1, 0.5 μg·mL^−1^), and lysophosphatidylcholine (d_7_-Lyso PC-18:1, 25 μg·mL^−1^). The mixed internal standards were stored at −20 °C until further use.

### 2.3. Determination of Storage Indicators of Quinoa during Storage

The lipase activity, fatty acid (FA) content, peroxide value (PV), and malondialdehyde (MDA) value were measured following China National Standards GB 5523-2008 [13], GB 15684-2015 [14], GB 5009.227-2016 [15], and GB 5009.181-2016 [16], respectively.

### 2.4. Lipidomic Analysis of Quinoa during Storage

#### 2.4.1. Sample Preparation and Quality Assurance

We added 200 mg of quinoa flour, 5 mL of MeOH/MTBE (1:3, *v*/*v*), and 10 μL of lipid internal standards to a glass tube and vortexed them for one min. The mixture was centrifuged at 5000 rpm for 20 min at 4 °C after being sonicated for 20 min. Then, the supernatant was gathered and concentrated in a vacuum concentrator at 25 °C. The extracted lipids were resuspended in 1 mL CHCl_3_/MeOH (1:1, *v*/*v*) and we continued to centrifuge them at 10,000 rpm for 10 min at 4 °C. Before testing, samples were passed through an organic filter membrane of 0.22 μm [17].

#### 2.4.2. Data Acquisition with UPLC-Q-Exactive Orbitrap MS/MS

The lipid composition of quinoa was analyzed with UPLC-Q-Exactive Orbitrap MS/MS (Thermo Fisher Scientific, Waltham, MA, USA). An ACQUITY UPLC HSS T3 column (2.1 × 50 mm, 1.8 μm, Waters, Milford, MA, USA) was used for chromatographic separation. The mobile phase was composed as follows: (A) water/MeOH/ACN (1:1:1, *v*/*v*/*v*) and (B) IPA/ACN (5:1, *v*/*v*) were both combined with 5 mM ammonium acetate. The following method was adopted for the separation program. The liquid phase gradient settings were as follows: 0–0.5 min, 20% B; 0.5–1.5 min, 20% to 40% B; 1.5–3 min, 40% to 60% B; 3 to 16 min, 60% to 95% B; 16 to 16.1 min, back to 20% B; kept at 20% B for 7 min. The flow rate was 0.3 mL·min^−1^ and the injection volume was 1 μL.

The scanning mode ranged from full MS to dd-MS^2^, which was performed in both positive ion mode and negative ion mode. The following mass spectrometry conditions were set: spray voltage 3.5 kV, sheath gas flow 35, auxiliary gas flow 10, drying gas temperature 350 °C, capillary temperature 320 °C, mass range from 200 to 1200 *m*/*z*, the resolution of full MS was 70,000, and the resolution of dd-MS^2^ was 17,500. The collision energy was 10, 30, and 50 eV [18]. We prepared quality control samples (QC samples) by combining equal amounts of each sample to monitor the stability of the system in real time and to assess data quality.

#### 2.4.3. Lipid Identification and Data Analysis

The following compound qualitative analysis methods were conducted. Exact mass measurement (<5 ppm), MS/MS fragmentation, LC retention time, and chemical compounds database query were used to identify lipids. Lipids were queried against multiple databases in LipidSearch^TM^ software (V4.2, Thermo Fisher Scientific, Waltham, MA, USA), MS-DAIL software (V4.00, http://prime.psc.riken.jp/Metabolomics_Software/MS-DIAL/index2.html, accessed on 25 September 2023.), and LIPIDMAPS (https://www.lipidmaps.org/, accessed on 25 September 2023.).

For quantitative analysis of compounds, Xcalibur 4.1 (Thermo Fisher Scientific, USA) was used to analyze the raw UPLC-Q-Exactive Orbitrap MS/MS data. Compound Discover^TM^ software 3.1 (Thermo Fisher Scientific, USA) was used to detect and pick the compound peaks based on the retention time and *m*/*z*. The semi-quantitative information about each component was then determined by comparing the peak area of each signal in the mass spectrum with an internal standard [19].

### 2.5. Statistical Analysis

The findings of each experiment are presented as mean ± standard deviation (SD), with each experiment being carried out in triplicate. The analysis of Student’s two-tailed *t*-test and ANOVA were used in SPSS 22.0 to test the statistical significance of the lipase activity, FA, PV, and MDA value. (SPSS, Inc., Chicago, IL, USA). At the *p* < 0.05 level, the results were considered statistically significant. Multivariate analysis, including principal component analysis (PCA) and partial least squares projection to latent structures-discriminate analysis (PLS-DA), were conducted by SIMCA 14.1 (Umetrics, Umea, Sweden) after treatment of pareto scaling [20]. In addition, the Kyoto Encyclopedia of Genes and Genomes (KEGG) pathway database was used to explore pathways related to lipid metabolism during the storage of quinoa flour.

## 3. Results and Discussion

### 3.1. Oxidative Stability during Storage

The surface area of enzymes and substrates is increased by flour products, which further triggers widespread lipid oxidation and degrades the nutritional value and storage stability of quinoa flours. Therefore, we assessed the storage stability of quinoa flour by measuring FA contents, PVs, MDA values, and lipase activities during storage. Among them, FAs are usually used to measure the degree of lipid hydrolysis, PVs and MDA values are used to measure the degree of oxidation of quinoa flour, and lipase is the key enzyme in the process of lipid hydrolysis.

**Figure 1:**
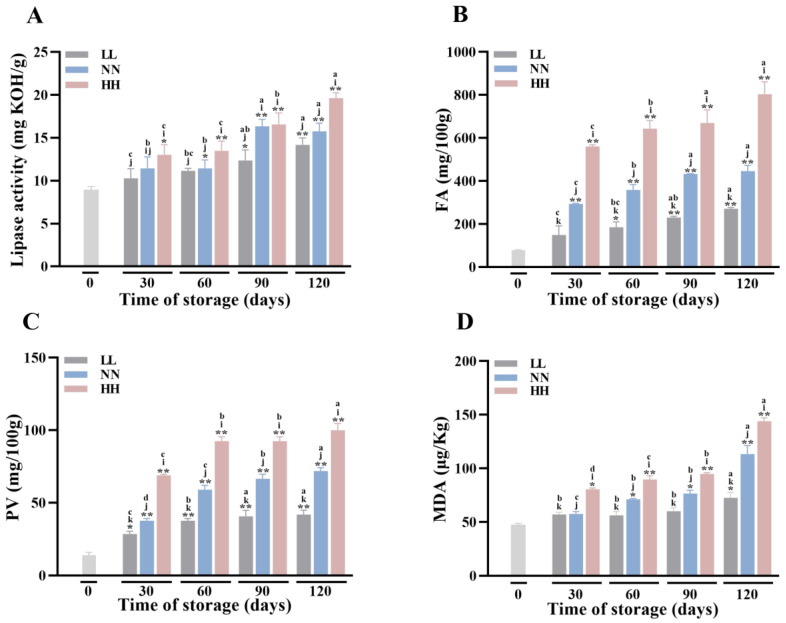
Oxidative stability during storage. The lipase activity (**A**), FA content (**B**), PVs (**C**) and MDA values (**D**) of quinoa flour with different treatments. Significant differences between all samples and 0 are shown by * *p* < 0.05, ** *p* < 0.01. Under the same storage time, significant differences (*p* < 0.05) among the samples from different storage conditions are shown by data bearing different letters (i, j, and k), respectively. Under the same storage condition, significant differences (*p* < 0.05) among the samples from different storage times are shown by data bearing different letters (a, b, c, and d), respectively.

Lipase activity is an important factor contributing to the loss of quinoa quality. The changes in lipase activity of quinoa stored at various conditions are shown in Figure 1A. It increased from 9.82 mg KOH/g at the beginning of storage to 14.83 (LL), 16.09 (NN), and 19.64 (HH) mg KOH/g at 120 days, respectively. It is clear that lipase activity increased with the prolongation of the storage time, indicating that quinoa lipids were continuously hydrolyzed during the storage process. At the same time, there were also significant differences between storage conditions, with lipase activity significantly higher (*p* < 0.05) in the HH condition than on the 0th day from the 30th day onwards, whereas no significant change in lipase activity occurred in the LL and NN conditions at this time. Lipase activity also started to increase significantly at 60 days of storage under NN conditions, and it was not until 90 days of storage that lipase activity under LL conditions started to change significantly. These results suggested that LL has a delaying effect on the hydrolysis of fat during the storage of quinoa flour, suggesting that low temperatures inhibit enzyme activity, whereas an increase in lipase activity is induced with increasing storage temperatures, which in turn accelerates lipid hydrolysis and oxidation.

During storage, the fat in quinoa is hydrolyzed to fatty acids by the lipase enzymes, leading to an increase in the fatty acid. So, FA was a crucial storage indicator that also had an impact on lipid oxidation [21]. As shown in Figure 1B, the FA values during 120 d of storage under LL, NN, and HH conditions were determined. As the samples were exposed for longer times, a significant increase (*p* < 0.05) in FA content was observed. It increased to more than three, five, and eight times the initial content at 120 days of storage, respectively. These results show that hydrolytic deterioration occurred in quinoa flour, which resulted in the production of free FAs. Compared with LL and NN conditions, the quinoa flour under the HH condition showed a significant increase in FAs (*p* < 0.01). The fatty acid values of LL and NN conditions were 64 percent and 40 percent lower than those of HH conditions when stored for up to 120 days. It can be seen that the hydrolysis of fats in quinoa can be slowed down under a low-temperature storage environment.

PV is a measure of the primary oxidation of fats because hydroperoxides are the main compounds formed during fat oxidation [22]. As shown in Figure 1C, the PVs of the samples increased from 15.23 to 42.26, 71.95, and 100.12 mg/100 g after 120 d of treatment in LL, NN, and HH conditions, respectively. The peroxide values of HH conditions were 58% (*p* < 0.01) and 28% (*p* < 0.01) higher than those of LL and NN conditions, respectively. As expected, HH conditions had the highest level of PV compared to LL and NN conditions. Although the PV continued to increase after 60 d, there was a significant slowing down of the rate of increase compared to the previous period. This is mainly because the value of hydroperoxide is in dynamic equilibrium. As the oxidation reaction proceeds, the peroxide is further oxidized to secondary oxidation products, which affects the shelf-life of quinoa and its related products in turn.

Hydroperoxides are primary oxidation compounds, which will produce various secondary oxidation products (including aldehydes, alcohols, acids, and ketones) after decomposition, thus producing unpleasant tastes and smells. Malondialdehyde (MDA) is a stable and abundant secondary end product which is considered to be one of the most significant indicators of the state of lipid peroxidation [23]. The accumulation of MDA indicates that lipids are oxidized and will cause damage to cells. Among them, the peroxidation of lipids significantly alters the physical properties of the lipid bilayer, which in turn affects its cytomembrane fluidity and permeability. And the accumulated MDA is thought to participate in the degradation of various biological functions by attaching to biomolecules such as proteins and nucleic acids [24]. Therefore, MDA was also used to assess quinoa lipid oxidation during storage in this study. The MDA contents of all studied samples as an indication of fat oxidation in quinoa are presented in Figure 1D. MDA values in LL and NN conditions were relatively lower during the first 30 days of storage, indicating that these flours did not initially display an oxidative rancidity. However, the MDA reached 80.66 μg/kg after 30 days of storage under HH conditions, which was significantly higher than that in LL and NN conditions (*p* < 0.05). Meanwhile, the malondialdehyde content at the end of storage was only 71.32 μg/kg under LL conditions, which was significantly lower than that under NN and HH conditions (*p* < 0.05), suggesting that high-temperature storage of quinoa flour increased the degree of membrane lipid peroxidation and the accumulation of MDA, which caused damage to cell membranes and cells. We also found that the changes in FAs and PVs were significantly greater than those in MDA throughout the storage process, which suggests that quinoa flour lipids were first hydrolyzed to produce fatty acids during storage, and then the fatty acids were further oxidized to produce primary and secondary oxidation products.

### 3.2. Global Lipidomics Analysis of Quinoa

Quinoa flour samples with three storage conditions at 0, 30, 60, 90, and 120 d were scanned three times by UPLC-Q-Exactive Orbitrap MS/MS in ESI^+^ and ESI^−^ modes. QC samples were injected in every 12 samples and followed by two blank samples at the beginning and end of the analysis batch.

**Figure 2:**
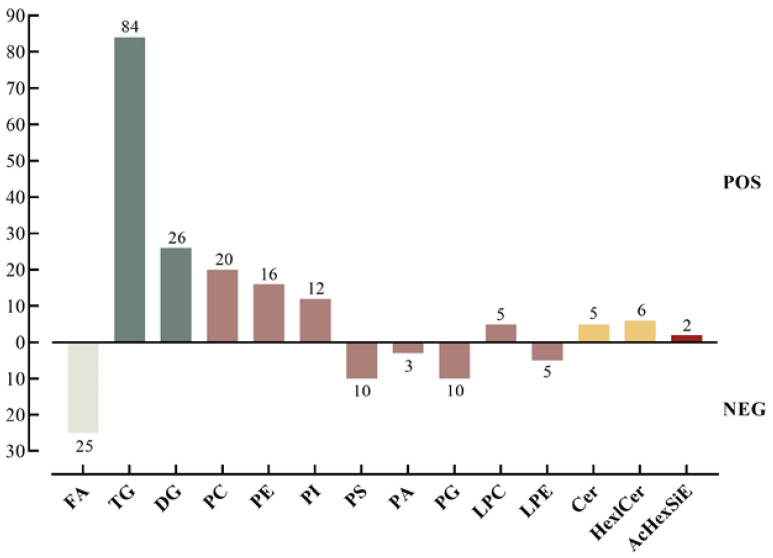
Identified lipid subclasses in quinoa flour. Lipids of the same colour belong to the same class.

The representative base peak chromatogram of the sample on 0 day is shown in Figure S1. There were 229 lipids found in Quinoa flour samples: 53 were found in the negative ion mode, and 176 were found in the positive ion mode. Quinoa lipids could be categorized into five categories by LIPID MAPS, including FA, GL, GP, SP, and ST. Furthermore, these five major classes could be further divided into 14 subcategories, including FA, TG, DG, PC, PE, PI, PS, PA, PG, LPC, LPE, Cer, Hex1Cer, and AcHexSiE (Figure 2). The most abundant subcategory was Triglyceride (TG) (84 lipids), followed by diglyceride (DG) (26 lipids) and fatty acyls (FAs) (25 lipids). The above results illustrate the diversity and complexity of quinoa lipids. In addition to TG ([M+NH4]^+^), DG ([M+NH4]^+^), PI ([M+NH4]^+^), and AcHexSiE ([M+NH4]^+^), others were detected as [M+H]^+^ or [M−H]^−^ adducts. Table S2 provides a complete list of 229 lipids with their lipid names, theoretical *m*/*z*, detection *m*/*z*, RT information, and mass accuracy.

### 3.3. Quantification and Changes in Quinoa Lipids during Storage

**Figure 3:**
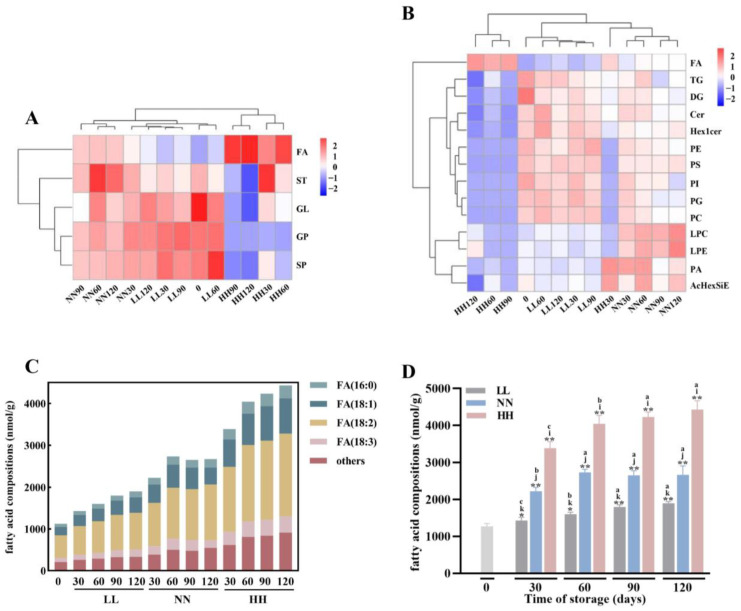
Changes in lipids in quinoa flour during storage. Changes in lipid classes in quinoa flour during storage (**A**). Changes in lipid subclasses in quinoa flour during storage (**B**). Composition of main fatty acids and their dynamic changes during storage (**C**). Changes in total fatty acid content (**D**). Significant differences between all samples and 0 are shown by * *p* < 0.05, ** *p* < 0.01. Under the same storage time, significant differences (*p* < 0.05) among the samples from different storage conditions are shown by data bearing different letters (i, j, and k), respectively. Under the same storage condition, significant differences (*p* < 0.05) among the samples from different storage times are shown by data bearing different letters (a, b and c) respectively.

In this study, an internal standard for isotopes was used to compare quantitative results. The sum of individual lipid contents was used to analyze and compare the content of similar or subclasses of lipids in quinoa flours, and heat maps (Figure 3A,B) were used to visualize the dynamic changes in quinoa lipid contents at various storage periods.

The most abundant lipids in quinoa were GLs and GPs, followed by SPs, STs, and FAs (Figure 3A). There were significant differences in various classes of lipids between unoxidized and oxidized quinoa samples. In the HH condition, the content of FAs was significantly increased from 1120.39 to 4432.24 nmol/g, and the contents of GLs and GPs were decreased from 320,040.07 and 8334.76 to 74,954.70 and 283.61 nmol/g, respectively. However, these differences were not significantly observed in LL and NN conditions. Lipid hydrolysis during quinoa storage is highly dependent on lipase and phospholipase [25]. TG is hydrolyzed to FA and glycerol by lipase and then releases DG or MG, which continue to be hydrolyzed. And GPs are more susceptible to oxidation because of more unsaturated bonds [26]. Therefore, FAs displayed a fluctuating upward trend, while GLs and GPs displayed a fluctuating downward trend during the storage period.

Next, we used a heat map to show the differences in lipid subclasses between fresh and stored quinoa flours at various storage stages (Figure 3B). The lipid profiles of quinoa flours change continuously during storage. Slight changes in the lipid composition of quinoa during the initial storage period may be attributed to the important role of antioxidant components such as superoxide dismutase and carotenoids in inhibiting quinoa lipid oxidation [27]. Later, with the increase in storage temperature and storage time, more changes took place in lipid compositions, and a significant change in lipid metabolite content was observed when stored in the HH condition for 120 days. This is due to the continuous involvement of lipids in quinoa flour in the corresponding metabolic reaction. Additionally, the oxidation process intensified with the degradation of antioxidant components in quinoa flour. The oxidation rate was also accelerated by lipid oxidation products with high oxidation capacities, such as free radicals and hydroperoxides [28].

TG exudates from the damaged cell membranes during pre-treatment processes, such as the shelling and milling of quinoa, which is further hydrolyzed by contact with previously dormant but highly active lipases in the endosperm layer and germ tissue [29]. We found 89 species of TG in quinoa in this study. The level of TG fluctuated up and down under LL and NN conditions, but finally decreased from 233,301.93 nmol/g to 203,824.95 nmol/g and 163,745.94 nmol/g, respectively. The decrease in TG content during storage was attributed to hydrolysis by lipase. And the level of TG increased, likely due to the action of glycerol-phosphate acyltransferase (the rate-limiting enzyme of TAG/GL synthesis) [30]. However, the content of TGs in quinoa flour under HH conditions was directly reduced to 59,573.33 nmol/g. The above results indicate that there was higher lipase activity under HH conditions, which increases the hydrolysis of TG. The FAs, produced by hydrolysis, are further oxidized to produce substances with odors as well as reducing the nutritional value of quinoa.

DG is one of the major lipid subclasses in organisms and serves as the second messenger for a variety of cellular activities, as well as participating in the biosynthesis of phospholipids such as PC, PE, and PS [20]. In this study, 26 species of DG were detected in quinoa. These changes followed a pattern that closely resembled that of TG. The results showed that the level of DG fluctuated up and down under LL and NN conditions, but finally decreased from 86,738.14 nmol/g to 54,125.28 nmol/g and 44,500.77 nmol/g, respectively. However, the content of DG in quinoa flour under HH conditions was directly reduced to 15,381.37 nmol/g. It is possible that DG accumulates due to the degradation of TG, whereas the decrease is due to hydrolysis by lipase.

Glycerophospholipids mainly include PAs, PCs, PEs, PGs, PSs, and PIs, which play a key role in maintaining cellular homeostasis and are the main structural components of cell membranes [31]. In total, 5 LPCs, 5 LPEs, 3 PAs, 20 PCs, 16 PEs, 10 PGs, 10 PSs, and 12 PIs were identified in the study. The abundance of PCs, PEs, PSs, PIs, and PGs was significantly reduced under HH conditions, which may be associated with phospholipase A in quinoa. Phospholipase A, which consists of phospholipase A1 and phospholipase A2, hydrolyzes the respective acyl chains to generate FA and lysophospholipids (LPLs) [32]. Similarly, these phenomena were obvious under HH conditions, but not obvious under LL and NN conditions. In conclusion, these findings show that quinoa’s lipid composition and content can be greatly impacted by storage conditions.

Ceramides and hexosylceramides are components of sphingolipids and glycosphingolipids, which play an important role in regulating cell differentiation, proliferation, apoptosis, aging, and other life activities [33]. And these molecules are important structural compounds that make up the lipid bilayer of the cell membranes. In particular, ceramide is involved in mitochondrial-mediated apoptosis [34]. The findings indicate a significant decrease in the content of Cer and Hex1Cer during storage under the HH condition, which indicates that storage conditions had a key influence on the degradation of Cer and Hex1Cer in quinoa. It is possible that the degradation of Cer may lead to mitochondrial dysfunction and apoptosis, which will affect the storage quality of quinoa.

We further analyzed the fatty acid composition of quinoa and identified 25 fatty acids in total. The unsaturated fatty acids of fresh quinoa flours were mainly composed of C18:1 oleic acid (17.68%), C18:2 linoleic acid (47.88%), and C18:3 linolenic acid (8.82%). Moreover, the proportion of saturated fatty acids was relatively low, and C16:0 palmitic acid (6.70%) was the main component (Figure 3C). FAs increased significantly (*p* < 0.05) in all quinoa samples during storage with the degradation of glycerolipids and glycerophospholipids (Figure 3D). However, the percentage of linoleic acid C18:2 was slightly reduced after accelerated storage for 120 d, and the percentages of palmitic acid C16:0 increased. A decrease in PUFAs and an increase in saturated fats (SFAs) were observed after quinoa was oxidized, which was consistent with the observations of Sun et al. [35]. An epidemiological study showed that intake of SFAs increased the risk of cardiovascular disease (CVD), but the intake of more PUFAs could slow down CVD [36]. The increase in SFAs and decrease in PUFAs were not beneficial to human health.

### 3.4. Significantly Different Lipids between Fresh and Stored Quinoa

**Figure 4:**
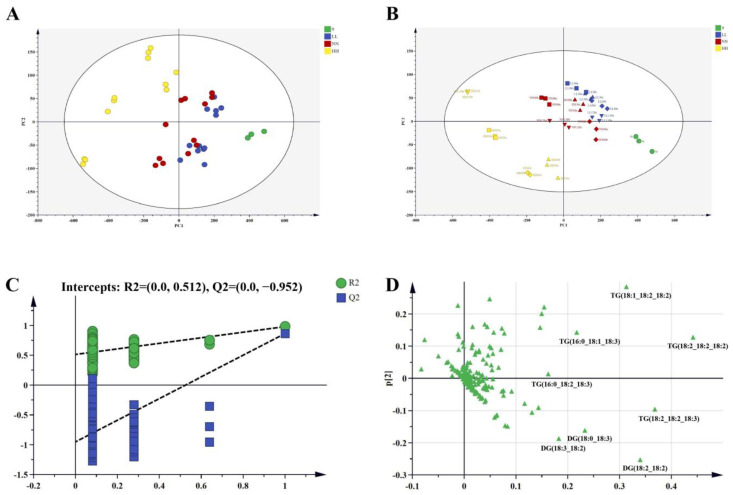
Multivariate statistical analysis of quinoa flour during storage. PCA score plot of lipid species (**A**). PLS-DA score plot (**B**). PLS-DA loading plot (**C**). Validation plots of PLS-DA (**D**).

To further explore changes in lipid composition, we analyzed and visualized lipidomics data during quinoa storage by multivariate statistical analysis. We performed PCA analysis of the lipidomic data to understand the metabolic differences among groups and the variations in the samples within groups, and explored the main factors contributing to differences during storage. Figure 4A shows the PCA score plot of quinoa lipid under different storage conditions. The distinction between 0 and HH conditions was clear, but the distances between LL and NN conditions were not clear. These findings indicated significant lipidome changes in HH conditions, and lipid oxidation was not significant under LL and NN conditions. However, the differences between samples of different oxidation stages were similar and could not be well distinguished. Therefore, the differences in quinoa lipid metabolism during different storage periods were additionally analyzed by PLS-DA, which resolved variations in the data more effectively than PCA. The PLS-DA score plot shows differences in lipid composition during quinoa oxidation. A clear separation was observed between unoxidized samples and other samples in HH conditions, but the differences among samples oxidized for 30 d, 60 d, 90 d, and 120 d in LL and NN conditions were small (Figure 4B). The score plot of the PLS-DA mode shows a good interpretation rate (R^2^X = 0.978 and R^2^Y = 0.945) and better predictability (Q^2^ = 0.864). Moreover, the robustness and predictability of the model were supported by the results of 200 permutation tests (Figure 4C). The intercepts of Q^2^ are expected to be below 0.00. According to the results, the PLS-DA model was reliable, predictive, and not overfit. The results confirmed that quinoa lipidomics profiles were significantly altered during storage in HH conditions.

To further elucidate the metabolic changes in quinoa during storage, we tried to explore the important differential lipid metabolites and associated pathways. Based on the PLS-DA model described above, we used the threshold *VIP* > 1 and *p* < 0.05 to screen for significantly different lipids during quinoa oxidation. The changes in these differential lipids during storage were visualized by the heatmap shown in Figure S2. A total of 90 significantly different lipids, including 40 TGs, 21 DGs, 2 PGs, 3 PSs, 6 PEs, 9 PIs, 8 FAs, and 1 HexlCer, were identified in PLS-DA. As a result of oxidation, eight FAs were significantly up-regulated, including FA (18:1) and FA (18:2). All of the remaining significantly different lipids were down-regulated, with PS (18:2_18:2) having the highest fold-change value (3.74), followed by PS (18:0_19:1) (3.47), PS (18:0_19:0) (3.47), and TG (4:0_16:1_18:2) (3.39) (Table S1). At the same time, the loading plot of PLS-DA (Figure 4D) shows that the lipid species of the TG and DG classes were the most important variables contributing to the discrimination between quinoa flour, which can be used to distinguish fresh quinoa flour from oxidized quinoa flour. The lipid content of quinoa changes during storage, which may have an impact on its composition, nutritional value, and function. Thus, it is necessary to investigate the specific functions and regulatory mechanisms of specific lipids during oxidation.

### 3.5. Correlation Analysis between Lipase Activity and Lipids

**Figure 5:**
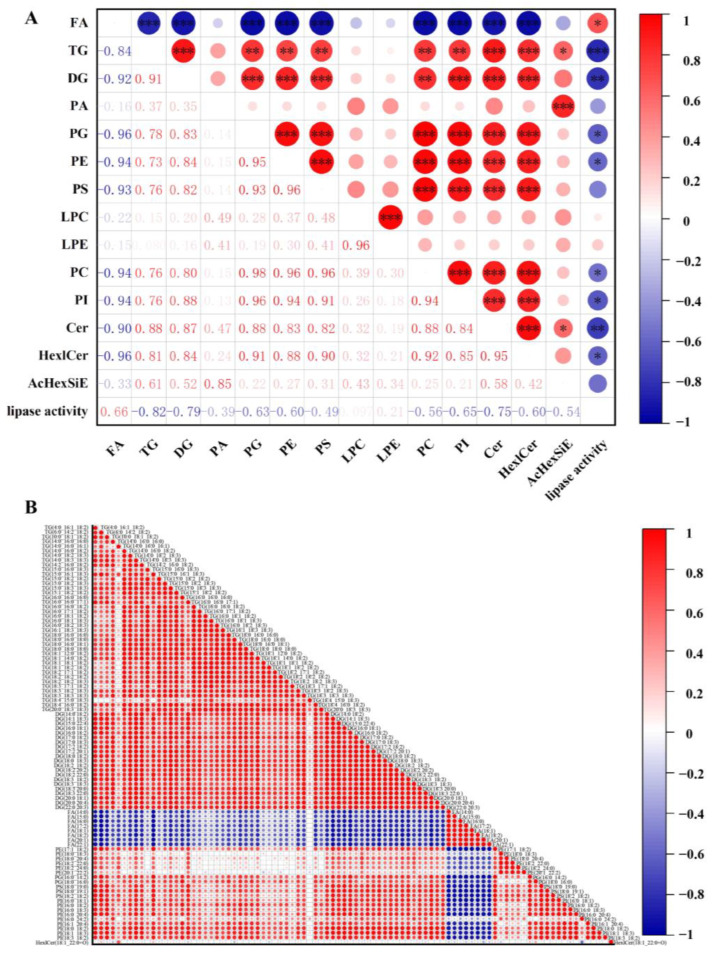
Lipid metabolism pathways during quinoa storage. Correlation analysis of lipids in quinoa flour. Correlation analysis of lipid subcategories and lipase activity (**A**). *** indicates *p* < 0.001, ** indicates *p* < 0.01, and * indicates *p* < 0.05. Correlation heat map analysis of 90 significantly different lipids in quinoa flour during storage (**B**).

We assessed the relationship between lipase activity and different subclasses of lipid content in quinoa during storage. The findings revealed a highly significant positive connection between lipase activity and FAs (R = 0.66) (Figure 5A). However, it was negatively correlated with most other lipids, especially TGs (R = −0.82) and DGs (R = −0.79). These results also indicated that TGs and DGs were hydrolyzed by lipase to produce FAs during the storage of quinoa flour. At the same time, we found that there were significant correlations between the different lipids in quinoa. For example, highly significant correlations were found between DGs and PCs (R = 0.80), PEs (R = 0.84), and PSs (R = 0.82), with some studies suggesting that DGs were involved in their synthesis [37]. Doblado-Maldonado et al. also found that the lipase activity was crucial to the deterioration of grain quality. It not only affects the lipid hydrolysis rancidity by lipase action [38], but also affects subsequent fatty acid degradation and oxidation. Therefore, enzyme inactivation during the pre-treatment of quinoa would contribute to its oxidative stability. Ling et al. used radio-frequency (RF) heating treatment to inactivate lipase activity in wheat germ and found that it could improve the physicochemical properties of wheat germ and enhance its lipid stability [39].

We conducted correlation heat map analysis on 90 lipids with substantial differences to discover the relationships between various lipids, and Pearson correlation coefficients were used to determine the correlation magnitude, which ranges from −1 to 1. Correlation size was assessed by the size of the circle, and negative or positive correlations were indicated by values closer to −1 or 1, respectively. We found that the majority of lipids were highly correlated with each other. Positive correlations were observed among lipids in the same subclasses and were stronger than the correlations between subclasses. As shown in Figure 5B, significant positive correlations were found between TGs and DGs as well as between PEs, PSs, PGs, and PIs, whereas they were all negatively correlated with FA. These were because the metabolism of both glycerolipids and glycerophospholipids produces FAs. Therefore, there was a complex relationship between significantly different lipids. The different lipids also interact with each other, which in turn affects the oxidation process of quinoa.

### 3.6. Lipid Metabolism Pathways during Quinoa Storage

**Figure 6:**
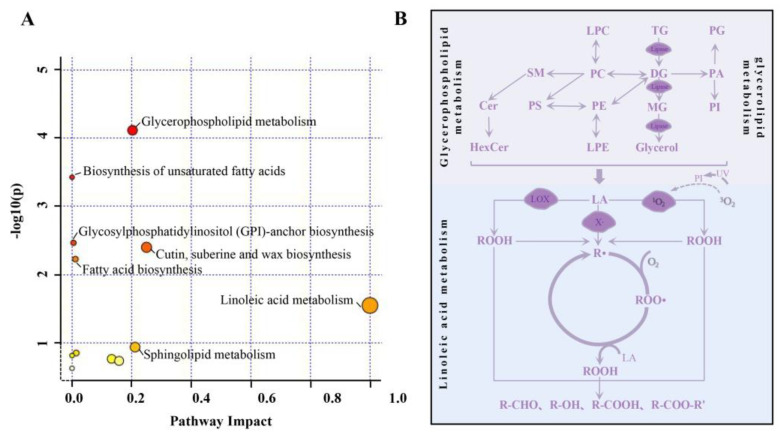
Metabolomic view map of important metabolic pathways of differentially expressed lipids in quinoa flour during storage (**A**). The *X*-axis represents pathway impact, and the *Y*-axis represents pathway enrichment. Potential pathways involved in the formation of important lipids in quinoa (**B**).

Metabolic pathway analysis is crucial for understanding the effect of storage time on the metabolic processes related to the lipid profiles of quinoa. In order to explore the lipid metabolic pathways associated with quinoa during storage, we mapped 90 species of significantly different lipids to the KEGG database to obtain the ID information. Moreover, MetaboAnalyst 5.0 was used to analyze metabolic pathways topologically. These significant lipids were mainly involved in 12 metabolic pathways, including the biosynthesis of fatty acids; the biosynthesis of unsaturated fatty acids; linoleic acid metabolism; glycerophospholipid metabolism; cutin, suberine, and wax biosynthesis; sphingolipid metabolism; GPI-anchor biosynthesis; glycerolipid metabolism; fatty acid elongation; inositol phosphate metabolism; the phosphatidylinositol signaling system; and fatty acid degradation. The results are shown as a bubble chart, and each bubble represents a metabolic pathway. Bubble size represents the enrichment value of the pathway and bubble color represent the influence value. As presented in Figure 6A, these significantly different lipids were mainly enriched for linoleic acid metabolism, glycerophospholipid metabolism, and keratin, cork, and wax biosynthesis. PEs, PIs, PSs, PGs, and FAs were involved in the three lipid metabolism pathways above.

Based on KEGG pathway analysis, Figure 6B shows the possible transformation of quinoa lipid molecules during storage. In the presence of phospholipase and lipase, hydrolyzed GP and GL could result in an increase in FAs, including FA (16:0), FA (18:1), and FA (18:2). During the hydrolysis of GL, TG, and DG, the major identified lipids is TG. At the same time, diacylglycerol acyltransferase (DGAT) could catalyze the covalent binding of DG with fatty acyl coenzyme A to produce TG. As mentioned in 3.3 above, the contents of TG and DG fluctuated up and down during the storage of quinoa. During the storage of quinoa, major lipids involved in the glycerophospholipid pathway include PE, PI, PS, and PG. DG could be involved in the synthesis of PE and PC as a lipid anchor in the presence of the corresponding phosphotransferases, while phospholipase C catalyzes the hydrolysis of PC and PE for the synthesis of DG [40]. In addition, lysophospholipid (LPC and LPE) could be generated via the cleavage of glycerophospholipid (PC and PE) molecules by phospholipase A1 or phospholipase A2, whereas lysophospholipid and acyl-coenzyme A could form glycerophospholipid in the presence of lysophospholipid acyltransferase. Among them, phospholipases play a critical role in the metabolism of phospholipids and the signaling of cells [41]. In the linoleic acid metabolism pathway, stored quinoa had a higher level of linoleic acid than fresh quinoa. GP and GL were degraded to produce linoleic acid (LA), which was subsequently oxidized to form primary oxidation products (ROOHs) under enzymatic and photosensitive oxidation involving lipoxygenase (LOX) and singlet oxygen (^1^O_2_), including 9-ROOH and 13-ROOH. In the auto-oxidation process, the methylene group of the double bond of unsaturated fatty acids removes an H and generates free radicals (R·). After entering the chain transfer stage, R· will generate peroxide radicals (ROO·) with the participation of oxygen due to instability, and ROO· will continue to react with LAs or other unsaturated fatty acids to generate new radicals (R·) and hydroperoxides (ROOHs) [42]. However, these hydroperoxides are unstable and can be further oxidized to form secondary oxidation products such as aldehydes, alcohols, acids, and esters (R-CHOs, R-OHs, R-COOHs, and R-COO-R’s), which in turn produce an irritating odor that affects the edibleness of quinoa. During the storage of quinoa, lipid oxidation is a significant reaction that leads to spoilage and deterioration. Nevertheless, the identification of these oxidation byproducts was not achieved in the present investigation. Meanwhile, the upregulation of FAs during quinoa storage leads to disturbances in the biosynthetic metabolism of keratins, spermine, and waxes, which reduces the protective effect of these substances on quinoa. The above findings in this research further confirmed the ideas in the relevant literature.

In conclusion, significant changes in lipids occurred during the storage of quinoa, involving 12 metabolic pathways and various enzymes. KEGG pathway analysis of these significantly different lipids in fresh and oxidized quinoa is helpful to explore the metabolic process of quinoa lipids during storage. Therefore, it is necessary to further study the metabolic pathways and enzymes related to this process. For example, it is necessary to identify the oxidation and degradation of FA products in order to provide a deeper understanding of these lipid metabolic pathways and reveal the functions of associated enzymes.

## 4. Conclusions

At present, the effects of storage conditions and storage time on lipase activity, lipid hydrolysis, and the rancidity and lipomics characteristics of quinoa were mainly studied. It turned out that low temperature and low humidity conditions considerably suppressed FA accumulation, lipase activity variation, and GL and GP hydrolysis and oxidation during storage. On the contrary, after 30 and 60 days of storage in NN and HH conditions, respectively, lipase activity, FA, PV, and MDA values started to increase significantly, which could reduce the nutritional value of quinoa flour and produce an irritating odor. At the same time, the long-term consumption of oxidized deteriorated lipids will also have an impact on human health. It will not only lead to unhealthy plasma lipids and cause cardiovascular diseases, but will also accelerate human aging due to the free radicals produced in the process of lipid oxidation. Simultaneously, the correlation analysis showed that lipase activity plays a key role in quinoa lipid metabolism. Therefore, we could improve its storage stability by reducing lipase activity. The above results provide a basis for the shelf-life of quinoa and its related products. In addition, the lipid composition of quinoa and the dynamic changes in lipid characteristics were clarified during storage. These achievements improve our understanding of quinoa’s oxidation mechanism during storage. It is helpful to further study quinoa in the future and promote the development and application of quinoa and its related products.

## Data Availability

Data are contained within the article.

## Supplementary Materials

The following supporting information can be downloaded at: https://www.mdpi.com/article/10.3390/foods12244434/s1, Figure S1: The representative base peak chromatogram of sample in 0 day in positive ion mode (A) and negative ion mode (B); Figure S2: Screening results of significantly different lipids in quinoa flour during storage; Table S1: Lipid compounds tentatively identified in Quinoa lipids extracts; Table S2: Identification of significantly different lipids during accelerated storage.
