# Supplementary material for: Evaluation of Lipidomics Profile of Quinoa Flour and Changes during Storage Based on Ultra Performance Liquid Chromatography Coupled with Quadrupole Exactive Orbitrap Mass Spectrometry
Source: Foods. 2023 Dec 11;12(24):4434. doi: 10.3390/foods12244434 (PMC10743080; doi:10.3390/foods12244434)
Supplement: Supplementary file 1 [file foods-12-04434-s001.zip › 7-Supporting Information.pdf]

Supporting Information

Evaluation of lipidomics profile of quinoa flour and changes during storage based on UPLC-Q-exactive orbitrap mass spectrometry

Yabo Ba[a], Rui Li[a], Jiayi Zhang[a], Liang Zou[a], Dingtao Wu[b*], Yichen Hu[a*]

[a] *School of Food and Biological Engineering, Key Laboratory of Coarse Cereal Processing (Ministry of Agriculture and Rural Affairs), Sichuan Engineering & Technology Research Center of Coarse Cereal Industrialization, Chengdu University, Chengdu 610106, Sichuan, People's Republic of China*

[b] *Institute for Advanced Study, Chengdu University, Chengdu 610106, Sichuan, China*

***Correspondence Author:**

wudingtao@cdu.edu.cn (D.-T. Wu), huyichen@cdu.edu.cn (Y.C. Hu)

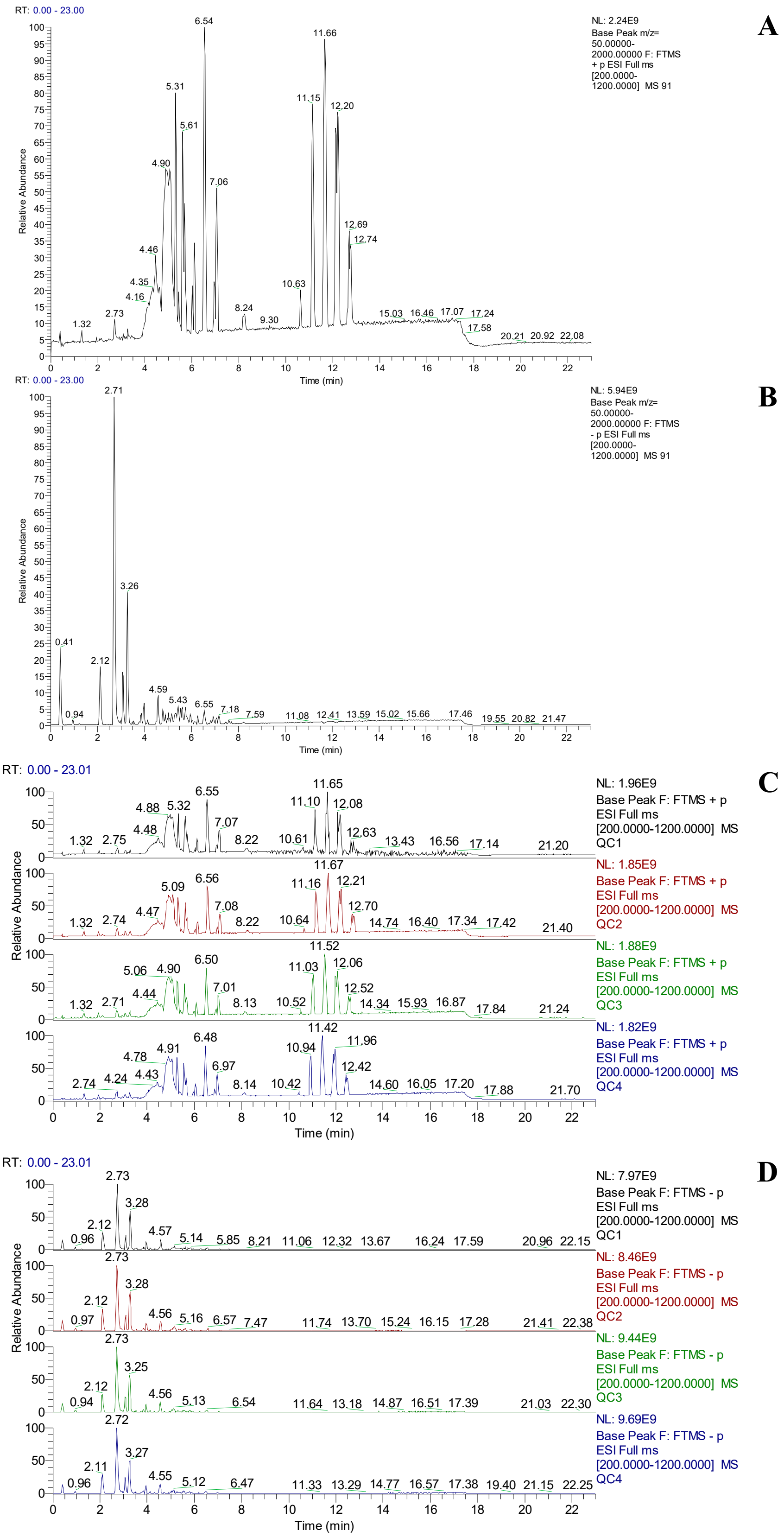

Figure S1. The representative base peak chromatogram of sample in 0 day in positive ion mode (A) and negative ion mode (B). The base peak chromatogram of QC in positive ion mode (C). The base peak chromatogram of QC in negative ion mode (D).

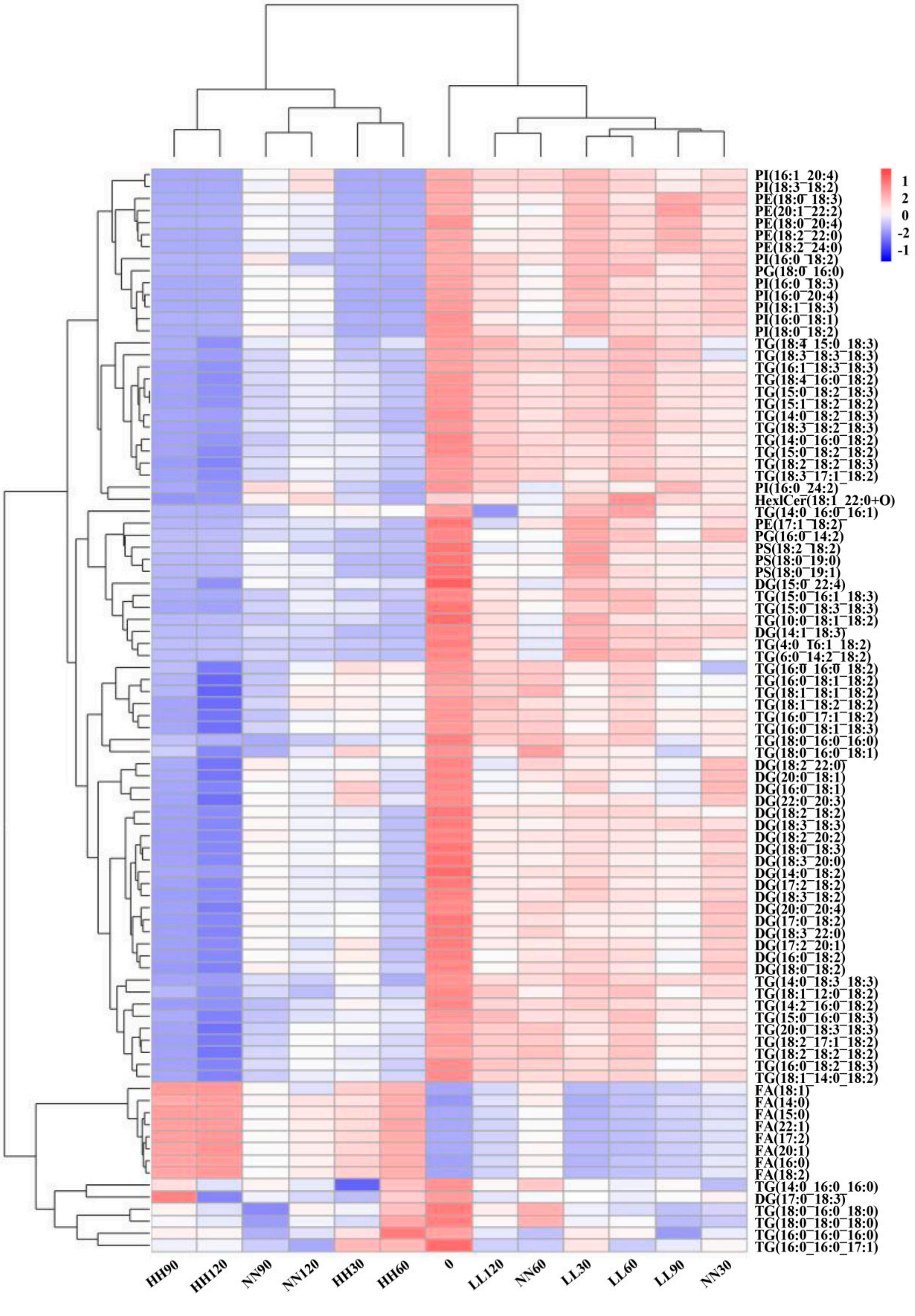

Figure S2. Screening results of significantly different lipids in quinoa flour during storage.

Table S2. Identification of significantly different lipids during accelerated storage.

Compound	VIP	P value	FC
TG(10:0_18:1_18:2)	1.68561	8.33E-06	3.20
TG(14:0_16:0_16:0)	1.28717	9.66E-04	1.43
TG(14:0_16:0_16:1)	1.12537	4.66E-03	1.95
TG(14:0_16:0_18:2)	1.42112	2.75E-04	1.82
TG(14:0_18:2_18:3)	1.36694	4.85E-04	2.22
TG(14:0_18:3_18:3)	1.29277	1.11E-03	2.29
TG(14:2_16:0_18:2)	1.38456	3.05E-04	1.80
TG(15:0_16:0_18:3)	1.21428	2.59E-03	1.64
TG(15:0_16:1_18:3)	1.43086	2.45E-04	2.50
TG(15:0_18:2_18:2)	1.36479	4.97E-04	1.87
TG(15:0_18:2_18:3)	1.29574	1.26E-03	1.91
TG(15:0_18:3_18:3)	1.59568	2.40E-05	2.50
TG(15:1_18:2_18:2)	1.29202	1.31E-03	1.91
TG(16:0_16:0_16:0)	1.07600	7.54E-03	1.26
TG(16:0_16:0_17:1)	1.69525	3.15E-06	1.62
TG(16:0_16:0_18:2)	1.20237	2.71E-03	1.56
TG(16:0_17:1_18:2)	1.22892	1.88E-03	1.57
TG(16:0_18:1_18:2)	1.14451	4.84E-03	1.45
TG(16:0_18:1_18:3)	1.21250	2.59E-03	1.50
TG(16:0_18:2_18:3)	1.35541	5.90E-04	1.72
TG(16:1_18:3_18:3)	1.11491	6.46E-03	1.82
TG(18:0_16:0_16:0)	1.46859	1.84E-04	1.41
TG(18:0_16:0_18:0)	1.42917	3.72E-04	1.24
TG(18:0_16:0_18:1)	1.22169	2.95E-03	1.38
TG(18:0_18:0_18:0)	1.41869	4.65E-04	1.30
TG(18:1_12:0_18:2)	1.38232	5.79E-04	1.72
TG(18:1_14:0_18:2)	1.38068	4.76E-04	1.73
TG(18:1_18:1_18:2)	1.02438	1.21E-02	1.41
TG(18:1_18:2_18:2)	1.19248	3.14E-03	1.44
TG(18:2_17:1_18:2)	1.17824	3.48E-03	1.64
TG(18:2_18:2_18:2)	1.05047	1.07E-02	1.45
TG(18:2_18:2_18:3)	1.20902	2.63E-03	1.71
TG(18:3_17:1_18:2)	1.18101	3.67E-03	1.81
TG(18:3_18:2_18:3)	1.23561	2.04E-03	2.04
TG(18:3_18:3_18:3)	1.12952	5.79E-03	1.89
TG(18:4_15:0_18:3)	1.16018	4.10E-03	1.73
TG(18:4_16:0_18:2)	1.26383	1.70E-03	1.87
TG(20:0_18:3_18:3)	1.08159	8.45E-03	1.54
TG(4:0_16:1_18:2)	1.51900	6.46E-05	3.39
TG(6:0_14:2_18:2)	1.43988	2.30E-04	3.28
DG(14:0_18:2)	1.74917	1.83E-06	2.34
DG(14:1_18:3)	1.50346	1.00E-04	3.16

DG(15:0_22:4)	1.85583	1.24E-07	2.15
DG(16:0_18:1)	1.40091	2.64E-04	1.82
DG(16:0_18:2)	1.49978	1.02E-04	1.88
DG(17:0_18:2)	1.59766	2.55E-05	1.92
DG(17:0_18:3)	1.16748	3.56E-03	1.74
DG(17:2_18:2)	1.59800	2.29E-05	2.11
DG(17:2_20:1)	1.54727	4.95E-05	2.04
DG(18:0_18:3)	1.62753	1.47E-05	1.95
DG(18:0_18:2)	1.44400	2.11E-04	1.83
DG(18:2_20:2)	1.51660	7.62E-05	1.94
DG(18:2_22:0)	1.44575	1.66E-04	1.62
DG(18:2_18:2)	1.58857	2.76E-05	1.94
DG(18:3_20:0)	1.63910	1.15E-05	2.04
DG(18:3_22:0)	1.55797	4.13E-05	1.88
DG(18:3_18:2)	1.46495	1.54E-04	1.99
DG(18:3_18:3)	1.55288	3.50E-05	2.10
DG(20:0_18:1)	1.44606	2.25E-04	1.55
DG(20:0_20:4)	1.41211	3.24E-04	1.80
DG(22:0_20:3)	1.38183	3.61E-04	1.66
FA(14:0)	1.23020	1.86E-03	0.38
FA(15:0)	1.04235	1.00E-02	0.33
FA(16:0)	1.10366	5.55E-03	0.38
FA(17:2)	1.00720	1.27E-02	0.25
FA(18:1)	1.01956	1.29E-02	0.38
FA(18:2)	1.06352	8.45E-03	0.42
FA(20:1)	1.00857	1.23E-02	0.36
FA(22:1)	1.02086	1.13E-02	0.39
PE(17:1_18:2)	1.63340	1.26E-05	3.35
PE(18:0_18:3)	1.04318	9.66E-03	2.09
PE(18:0_20:4)	1.32821	6.74E-04	2.52
PE(18:2_22:0)	1.05700	8.70E-03	2.21
PE(18:2_24:0)	1.01630	1.17E-02	2.21
PE(20:1_22:2)	1.02003	1.18E-02	2.27
PG(16:0_14:2)	1.46188	1.52E-04	3.23
PG(18:0_16:0)	1.08698	7.48E-03	2.22
PI(16:0_18:1)	1.38063	3.84E-04	2.54
PI(16:0_18:2)	1.01665	1.36E-02	2.27
PI(16:0_18:3)	1.09818	6.16E-03	2.26
PI(16:0_20:4)	1.20661	2.43E-03	2.19
PI(16:0_24:2)	1.21786	2.54E-03	2.14
PI(16:1_20:4)	1.04799	1.02E-02	1.97
PI(18:0_18:2)	1.31191	8.43E-04	2.42
PI(18:1_18:3)	1.27304	1.26E-03	2.25
PI(18:3_18:2)	1.09741	6.46E-03	2.05

PS(18:0_19:0)	1.62146	1.37E-05	3.47
PS(18:0_19:1)	1.65878	6.83E-06	3.47
PS(18:2_18:2)	1.62478	1.30E-05	3.74
HexlCer(18:2_22:0+O)	1.17413	2.71E-03	1.83
